# Nasopharyngeal Migration of Coils Following Embolization of a Traumatic Pseudoaneurysm of the Internal Carotid Artery: A Case Report

**DOI:** 10.7759/cureus.58121

**Published:** 2024-04-12

**Authors:** Jonathan Pimiento Figueroa, Mariana Escobar Howard, Pablo Escudero Pineda, Sebastian Orozco Arteaga

**Affiliations:** 1 Radiology, Servicios de Salud San Vicente Fundación, Medellín, COL; 2 Otolaryngology, Head and Neck Surgery, Universidad de Antioquia, Medellín, COL; 3 Interventional Neuroradiology, Servicios de Salud San Vicente Fundación, Medellín, COL; 4 Otolaryngology, Head and Neck Surgery, Hospital Universitario de San Vicente Fundación, Medellín, COL

**Keywords:** extrusion of embolization coils, endovascular therapy, internal carotid artery, traumatic pseudoaneurysm, epistaxis

## Abstract

Post-traumatic pseudoaneurysms of the internal carotid are a rare but potentially fatal cause of epistaxis; they are associated with fractures of the base of the skull with involvement of the carotid canal. Endovascular management is the preferred therapeutic strategy, with optimal long-term results and low complication rates. Complications may include thromboembolic events, infarction of perforating arteries, and rupture of the pseudoaneurysm. We present a case of a 28-year-old male with a post-traumatic pseudoaneurysm of the internal carotid who was managed with endovascular therapy. A late complication was the extrusion of the embolization material into the nasal cavity and nasopharynx, which was safely and effectively treated through endovascular and endoscopic approaches.

## Introduction

Post-traumatic pseudoaneurysms of the internal carotid artery (ICA) represent less than 1% of all intracranial aneurysms [1]. While uncommon, they can lead to potentially lethal posterior epistaxis, with mortality rates varying from 30% to 50% [2,3]. These injuries are often linked to skull base fractures impacting the carotid canal. In certain cases, the injury may not manifest until nosebleeds become the primary symptom. A multidisciplinary approach to patient care is crucial, with treatment decisions tailored to the aneurysm's location, size, and the patient's clinical condition. Endovascular embolization is widely accepted due to advancements in techniques providing favorable long-term outcomes. Late complications such as embolization material migration occur in only 2-6% of cases [4]. Here, we describe a 28-year-old male experiencing severe epistaxis due to a post-traumatic pseudoaneurysm of the left ICA, where delayed migration of the embolization material toward the nasal cavity and nasopharynx occurred.

## Case presentation

A 28-year-old man with a history of severe head trauma nine months ago sought consultation for recurrent episodes of self-limited spontaneous epistaxis that began approximately three weeks after the mentioned trauma. Upon admission, blood tests revealed a hemoglobin level of 4.4 mg/dl.

**Figure 1:**
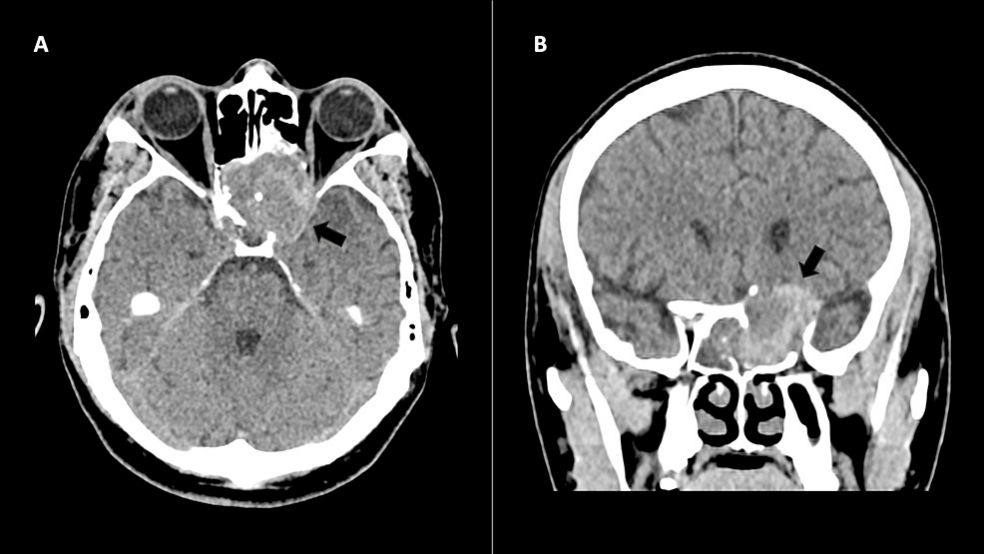
Skull CT. A: axial B, coronal. Heterogeneous mass (arrows) with hyperdense areas, located in the left sphenoid sinus, showing remodeling of the sinus walls, posterior ethmoid cells, medial wall of the left orbit, and a bone defect in the base of the skull (anterior cranial fossa and medial cranial fossa).

Initially, a skull computed tomography (CT) revealed a heterogeneous expansile lesion in the left sphenoid sinus, with areas of increased density and remodeling of the sinus walls, the posterior ethmoid cells, and the medial orbit wall on that side. The lesion was also linked to a skull base bone defect (Figure 1).

**Figure 2:**
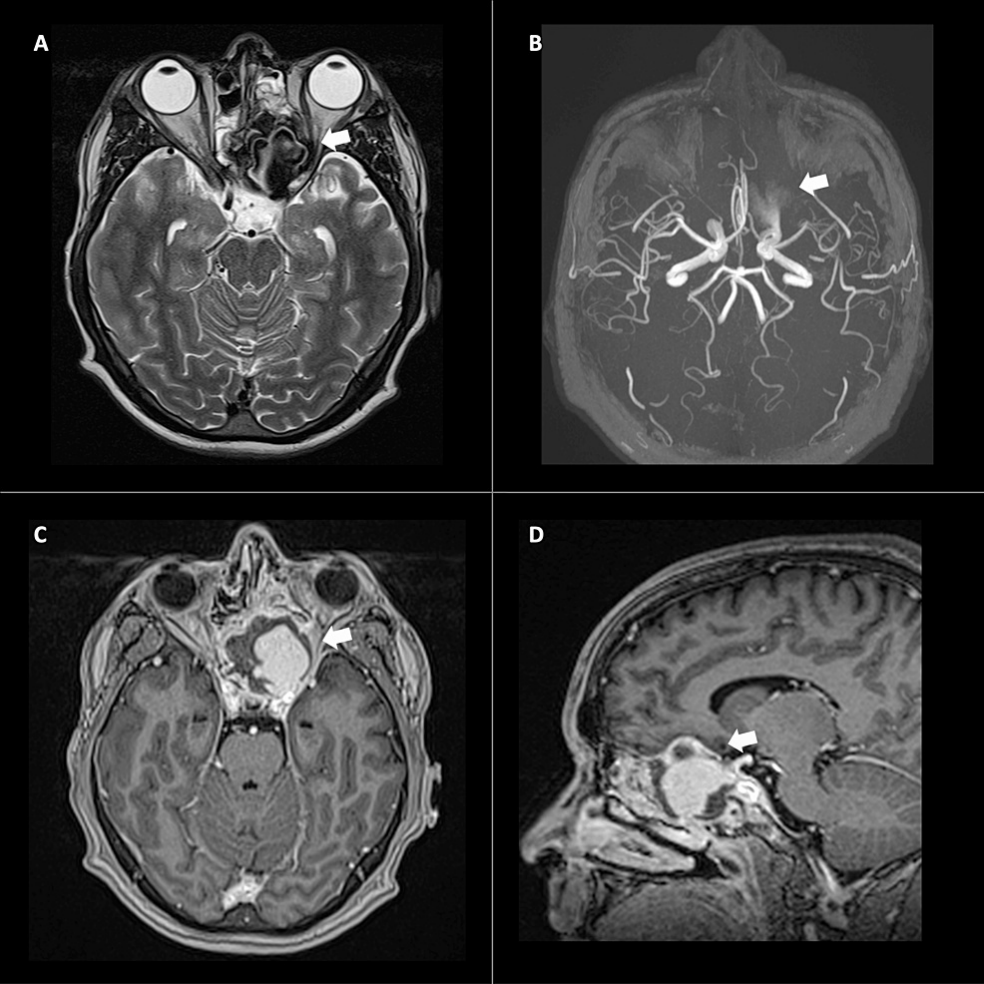
Contrasted MRI of the brain. A. Axial T2-weighted. A well-defined heterogeneous lesion is present in the left sphenoid sinus, showing partial signal void connecting to the ophthalmic portion of the left ICA. B. TOF MPR. The lesion is partially defined by partial signal void, likely caused by slow flow. C. T1-weighted post gadolinium axial and D. sagittal. The lesion shows avid enhancement with contrast, indicating continuity with the left ICA. A diagnosis of pseudoaneurysm of the ophthalmic segment of the left ICA was made. ICA: Internal carotid artery

Subsequently, a contrast-enhanced magnetic resonance imaging (MRI) of the brain revealed an expansive lesion located in the left sphenoid sinus. This lesion connects with the left ICA in its ophthalmic segment, showing a slight signal voiding in the weighted sequences T1 and T2, along with time-of-flight (TOF). The lesion exhibits enhancement in the contrast sequences, confirming the presence of a post-traumatic pseudoaneurysm in the left ICA (Figure 2).

**Figure 3:**
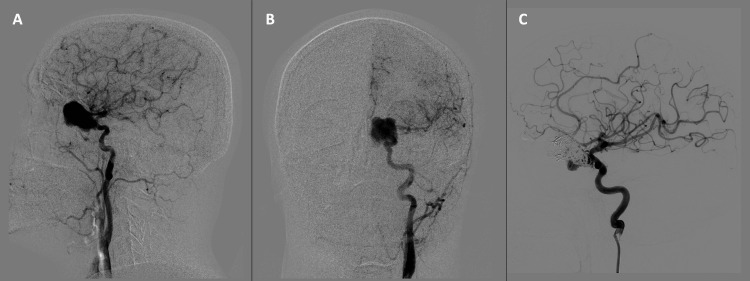
Digital subtraction angiography (DSA). A-B Saccular image located at the medial aspect of the ophthalmic segment of the left ICA. Left ICA pseudoaneurysm. C. Left ICA pseudoaneurysm exclusion with 10 coils. ICA: Internal carotid artery

**Figure 4:**
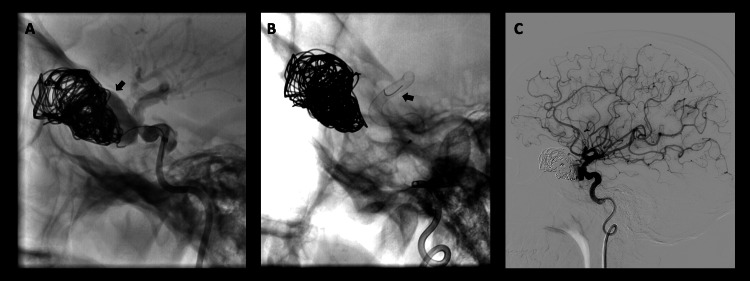
Angiography – Digital subtraction angiography. A. Coils inside the pseudoaneurysm show contrast passage to the cavity not occupied by the embolization material (arrow). B. Flow diverter in the left ICA (arrow). C. Control after the placement of flow diverter without complications. ICA: Internal carotid artery

A cerebral angiography revealed a giant pseudoaneurysm resulting from a rupture in the ICA's ophthalmic segment (Figure 3). To control bleeding, emergency treatment began with embolization of the pseudoaneurysm involving the placement of 10 coils (Figure 3). Subsequently, a flow diverter stent was inserted a week later to successfully exclude the aneurysm (Figure 4). Following these procedures, there was a demonstrated redirection of blood flow (Figure 4).

**Figure 5:**
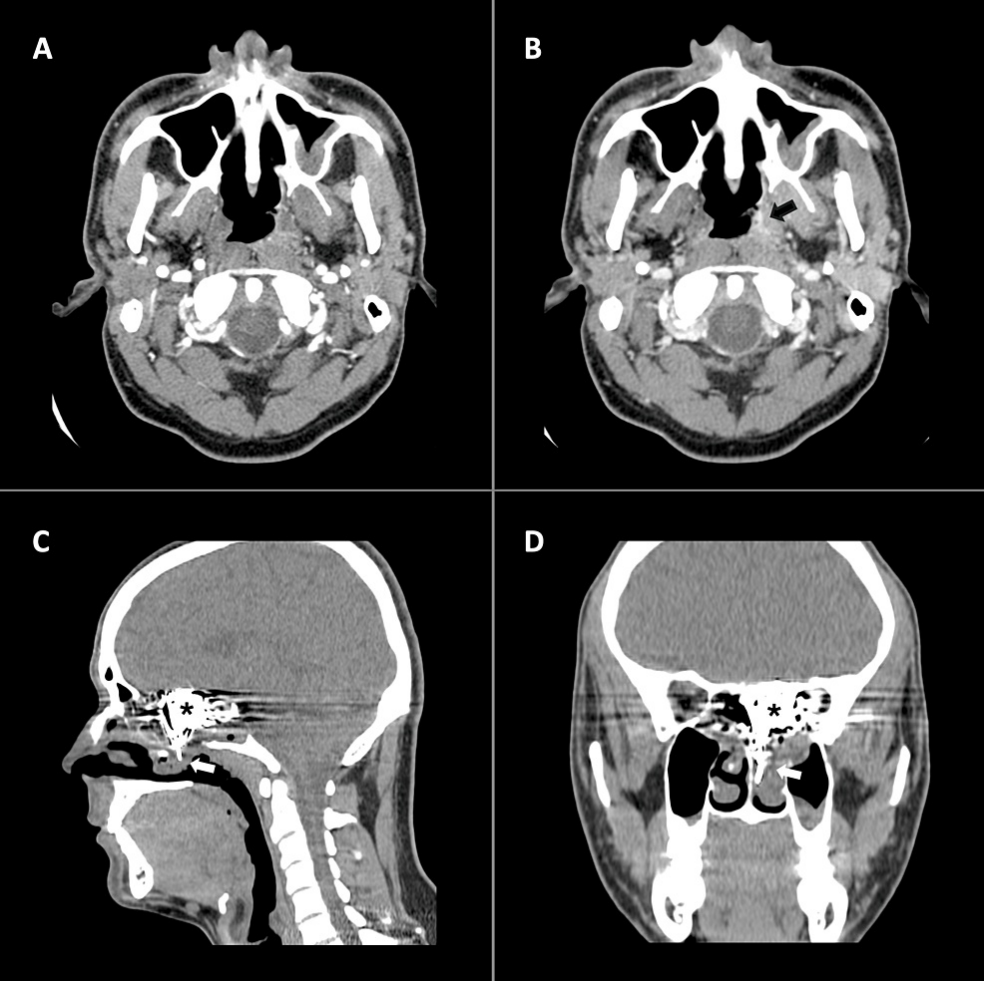
Angiotomography of the skull and neck. A-B. Venous phase. Axial. Contrast extralumination near the lateral aspect of the left choana (Arrow) extending to the tubal torus, active bleeding likely from branches of the left sphenopalatine artery. C-D. Without contrast phase. Sagittal-Coronal. Embolization material in left ICA pseudoaneurysm (asterisk). Extrusion of the embolization material extending to the left nostril (white arrow). ICA: Internal carotid artery

**Figure 6:**
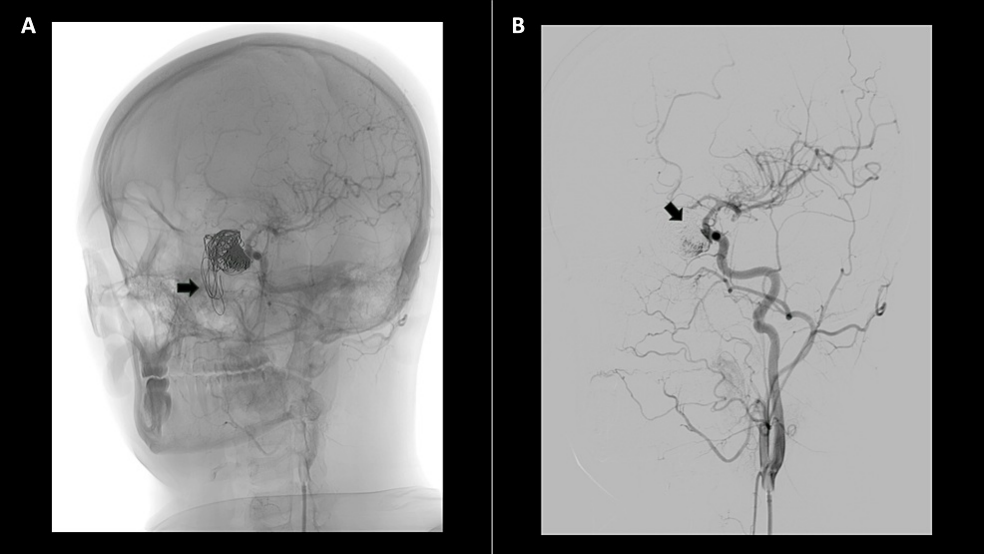
Angiography - Digital subtraction angiography A. Extrusion of coils into the left nostril (arrow) B. Adequate exclusion of left ICA pseudoaneurysm. ICA: Internal carotid artery

Twenty days post initial consultation, the patient visits the emergency room following a fresh bout of severe epistaxis. Angiotomography (CTA) reveals active bleeding that, due to its location, suggests an origin in branches of the sphenopalatine artery (Figure 5). A result of the previous endovascular management of the pseudoaneurysm, was also observed projecting onto the nasal cavity (Figure 5). A new embolization of the left pterygopalatine segment was carried out with 300-500 Mcs PVA microparticles and two coils. Pre-embolization imaging showed coil migration to the left nasal fossa and successful exclusion of the pseudoaneurysm from prior treatment (Figure 6).

**Figure 7:**
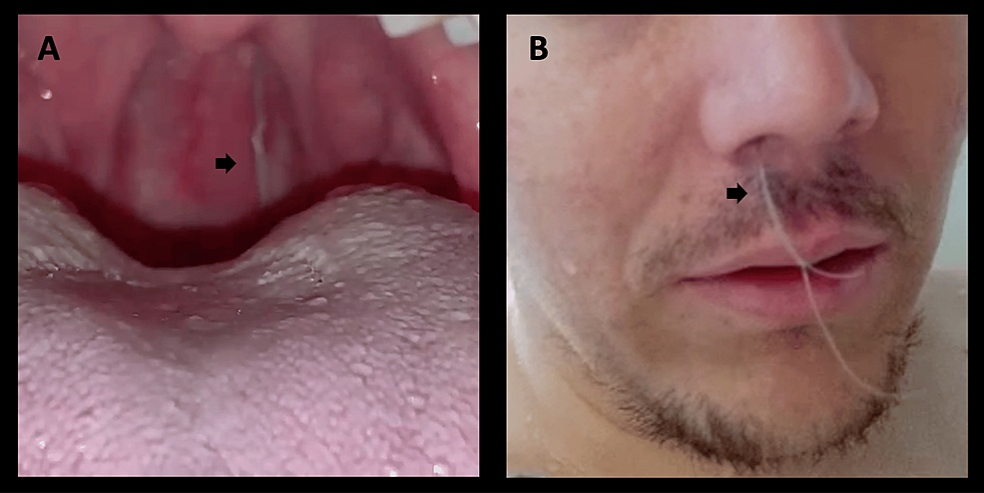
Coils extrusion A. Coil extrusion in the oropharynx (arrow). B. Photo of the patient with coils protruding through the nose (arrow).

**Figure 8:**
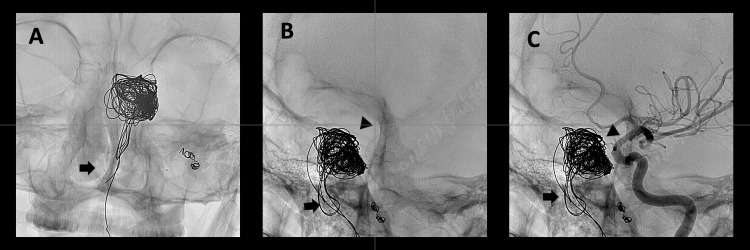
Extrusion of the embolization material - Angiography A-B coronal – sagittal. Extrusion of the embolization material (coils) into the nasopharynx and oropharynx (arrow). Correctly positioned flow diverter (arrowhead). C sagittal. Extrusion of coils into the nasopharynx and oropharynx (arrow). Successful exclusion of the left ICA pseudoaneurysm, with no connection between the ICA and the coil-containing sac (arrowheads). ICA: Internal carotid artery

Twenty-four hours post embolization of the pterygopalatine segment, the patient experienced another episode of significant epistaxis. Otorhinolaryngology then conducted a nasosinuscopy to manage the epistaxis endoscopically. During the procedure, the material from previous embolizations was observed to have extruded into the nasal fossa, with no active bleeding. Subsequently, the material was spontaneously expelled through the nasal passages (Figure 7). These findings were confirmed in a follow-up angiography, showing proper exclusion of the pseudoaneurysm and no communication between the internal carotid and the coil sac (Figure 8).

**Figure 9:**
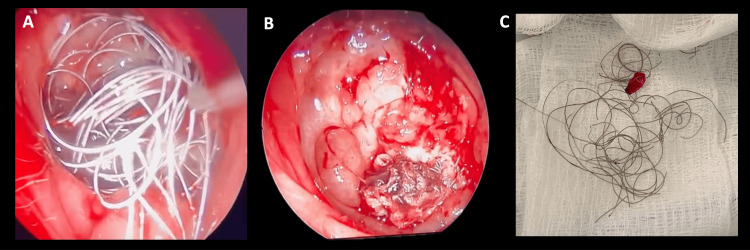
Transnasal endoscopic removal of the extruded material. A. Photograph of the extruded embolization material. B. Photograph of skull base at the end of extraction. C. Multiple coils extruded.

**Figure 10:**
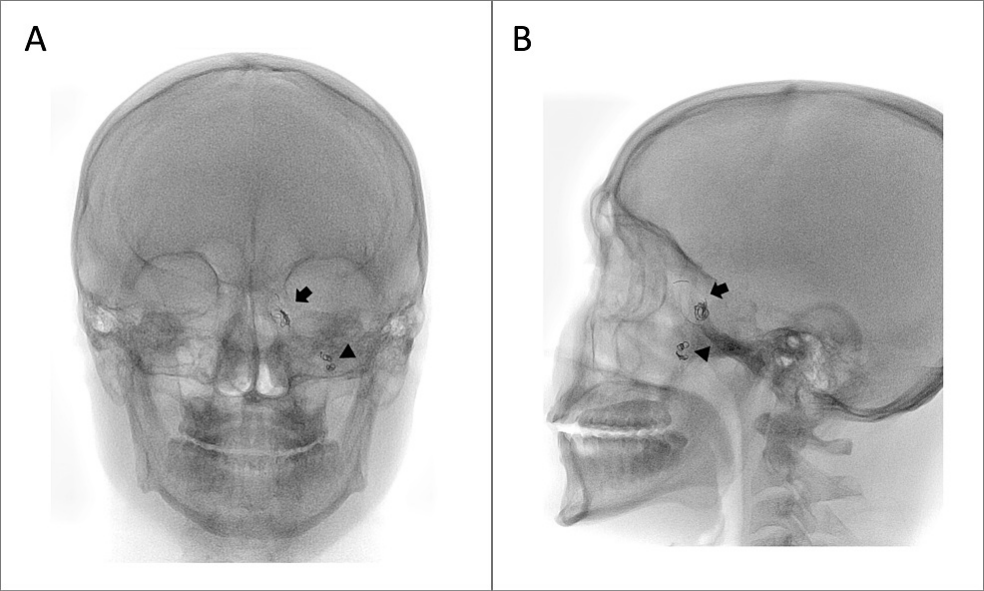
Residual embolization material - Fluoroscopy A - B, anteroposterior – side views. Residual embolization material (coils) in the pseudoaneurysm sac (arrows) and in the left pterygopalatine segment (arrowheads).

After a comprehensive evaluation, based on both surgical and imaging findings, which showed complete occlusion of the pseudoaneurysm with a line of healing, along with extrusion of the embolization material toward the nasal cavity, nasopharynx, and hypopharynx. It was decided to perform an endoscopic extraction of the remaining material. The procedure was carried out through endoscopic paranasal sinus surgery by otorhinolaryngology. During the intervention, a bone defect of the anterior skull base was observed, as well as fibrotic scar tissue and multiple coils remains. The latter were removed endoscopically: some were cut and removed, while those embedded in the scar tissue were left in position, being cut flush with the tissue to prevent future trauma to the mucosa and eventual bleeding (Figure 9). Imaging control was performed demonstrating the remaining embolization material (Figure 10). During the post-treatment follow-up, it was noted that the patient did not experience any new episodes of epistaxis.

## Discussion

Nosebleeds are a common reason for consultation in emergency departments and are often managed conservatively, however, there are cases where this approach is insufficient [5]. Situations that do not resolve with conventional management or that are life-threatening require rapid diagnosis and immediate intervention. Therapeutic options include bedside procedures such as tamponade or cautery, endoscopic surgery, and/or endovascular management [6]. Pseudoaneurysms of the ICA are rare and can be caused by various factors such as transsphenoidal surgery, closed trauma, open trauma, inflammatory processes, radiation, vascular dissection, and collagen diseases [7,8].

The onset of post-traumatic ICA pseudoaneurysms typically occurs later, usually appearing approximately three weeks following the traumatic incident [2]. Nevertheless, this latency period may prolong up to eight months [9], leading to challenges in their initial detection through scans. Common indicators of these pseudoaneurysms encompass Maurer's triad, characterized by unilateral amaurosis, orbital fracture, and significant epistaxis, along with other visual impairments, carotid bruit, and fractures in the anterior skull base [3].

Although traditional angiography is still considered the "gold standard" for diagnosing ICA pseudoaneurysms, less invasive methods like angioresonance (MRA) or CTA are often preferred early on due to the valuable diagnostic information they provide [2]. Fontela and colleagues have introduced a diagnostic algorithm that suggests utilizing CTA/MRA when identifying bone lesions near the ICA and recommend repeating the study within 3 to 4 weeks to account for the latency period [3]. In terms of treating ICA pseudoaneurysms, several approaches have been employed, including surgically excluding the pseudoaneurysm, surgically ligating the cervical ICA, and occluding the ICA both proximally and distally to the pseudoaneurysm with extracranial to intracranial arterial bridging. However, endovascular procedures are often favored for their ability to lower the risk of neurological complications. Vascular procedures available include embolization with coils, bare metal stents, stents covered with autogenous vein grafts, stent grafts, and flow diverters [10].

Complications related to the endovascular approach can vary. In the short term, these may involve thromboembolic events, infarction in perforating arteries or side branches, vessel or aneurysm rupture, and delayed intraparenchymal hemorrhage, with less frequent occurrences of device migration or extrusion. Similarly, in the long term, there is a possibility of lesion recurrence and in-stent stenosis [11-13]. The extrusion of embolization material is an infrequent occurrence. Although with time, the walls of the pseudoaneurysm typically mature enough to secure the embolization material (coils) in place, there are instances where displacement can occur [2]. Depending on the anatomical location, the extrusion may be directed toward the sinus cavity or even the skull [4,5,8]. While the extrusion of material can lead to severe bleeding episodes, in the case we are discussing, this was averted due to the exclusion of the pseudoaneurysm with a flow diverter stent. Consequently, at the time of extrusion, there was no connection between the ICA and the pseudoaneurysm, enabling the partial removal of the extruded material by the otolaryngology team without subsequent complications.

## Conclusions

Post-traumatic pseudoaneurysms of the ICA are a rare but potentially fatal condition. Therefore, in cases of craniofacial fractures near this artery and suggestive clinical signs, it is crucial to maintain a high level of vigilance and suspicion. Delayed extrusion of the embolization material is an even rarer occurrence. However, as demonstrated in the case presented, although it is a rare complication of endovascular treatment, it can arise under specific circumstances, especially due to the absence of a true wall to contain the material and the size of the pseudoaneurysm. Managing these cases in specialized centers with a multidisciplinary approach will always be the optimal way to address them.
